# Exploring sex differences in blood-based biomarkers following exhaustive exercise using bioinformatics analysis

**DOI:** 10.5114/biolsport.2024.132998

**Published:** 2024-01-02

**Authors:** Julia C. Blumkaitis, Natalia Nunes, Tilmann Strepp, Aleksandar Tomaskovic, Mario Wenger, Hannah Widauer, Lorenz Aglas, Perikles Simon, Thomas Leonhard Stöggl, Nils Haller

**Affiliations:** 1 Department of Sport and Exercise Science, University of Salzburg, Salzburg, Austria; 2 Genetics Division, Department of Morphology and Genetics, Universidade Federal de São Paulo, São Paulo, Brazil; 3 Department of Biosciences and Medical Biology, University of Salzburg, Salzburg, Austria; 4 Department of Sports Medicine, Rehabilitation and Disease Prevention, Johannes Gutenberg University of Mainz, Mainz, Germany; 5 Red Bull Athlete Performance Center, Salzburg, Austria

**Keywords:** cfDNA, Cytokines, Exercise immunology, Exercise physiology, K-means clustering

## Abstract

This study examined the acute effects of exercise testing on immunology markers, established blood-based biomarkers, and questionnaires in endurance athletes, with a focus on biological sex differences. Twenty-four healthy endurance-trained participants (16 men, age: 29.2± 7.6 years, maximal oxygen uptake (${\dot{\text{V}}\text{O}}_{2\max}$): 59.4 ± 7.5 ml · min^−1^ · kg^−1^; 8 women, age: 26.8 ± 6.1 years, ${\dot{\text{V}}\text{O}}_{2\max}$: 52.9 ± 3.1 ml · min^−1^ · kg^−1^) completed an incremental submaximal exercise test and a ramp test. The study employed exploratory bioinformatics analysis: mixed ANOVA, k-means clustering, and uniform manifold approximation and projection, to assess the effects of exhaustive exercise on biomarkers and questionnaires. Significant increases in biomarkers (lymphocytes, platelets, procalcitonin, hemoglobin, hematocrit, red blood cells, cell-free DNA (cfDNA)) and fatigue were observed post-exercise. Furthermore, differences pre- to post-exercise were observed in cytokines, cfDNA, and other blood biomarkers between male and female participants. Three distinct groups of athletes with differing proportions of females (Cluster 1: 100% female, Cluster 2: 85% male, Cluster 3: 37.5% female and 65.5% male) were identified with k-means clustering. Specific biomarkers (e.g., interleukin-2 (IL-2), IL-10, and IL-13, as well as cfDNA) served as primary markers for each cluster, potentially informing individualized exercise responses. In conclusion, our study identified exercise-sensitive biomarkers and provides valuable insights into the relationships between biological sex and biomarker responses.

## INTRODUCTION

Assessing the physiological response to exercise is important for athletes, coaches, and sports scientists to optimize training load management and improve performance. Acute and strenuous exercise initiates stress and might lead to muscular damage, immune response, and inflammatory processes [1–3]. Exercise-induced responses are commonly monitored through objective monitoring tools (e.g., biomarkers) [4, 5]. Established blood-based biomarkers (for simplicity, we will continue to refer as biomarkers), such as blood lactate (LA) and creatine kinase (CK), are widely used but have limited informative value in the acute exercise setting when used solely in the absence of other biomarkers. Therefore, further exercise-sensitive biomarkers or biomarker panels have been suggested to comprehensively assess the physiological exercise response [6, 7]. These include, for instance, cytokines (e.g., tumor necrosis factor-alpha (TNF-α)-, interleukin-6 (IL-6)) or markers of aseptic inflammation (such as cell-free DNA (cfDNA)) which have been proposed as reliable markers of exercise sensitivity [3, 8–13]. Previous studies have found that IL-6 concentrations depend on exercise intensity and increase immediately after exercise, possibly up to 100-fold after a marathon race [11, 14, 15]. In a professional soccer setting, cfDNA showed promising results at rest [16] and directly after a soccer game, where median cfDNA increased 23-fold, and correlated with the total distance covered [8].

However, athlete responses to exercise are highly intra- and inter-individual as biomarkers are influenced by training status, exercise duration and intensity [7, 17, 18]. To date, there is limited information on how acute exercise responses are influenced by biological sex. This limitation arises in part from selective recruitment of men in previous studies, due to the complexity of hormonal variations and unique responses associated with the female menstrual cycle [19, 20].

Lobo et al. showed that a single bout of strenuous aerobic exercise induced similar responses in both sexes in leukocyte counts and cytokine levels [20]. In contrast, Bernadi et al. observed higher baseline concentrations in certain cytokines (i.e., IL-6, IL-1β, and TNF-α) in men compared to women but found no association with testosterone levels, implying that hormone levels may not fully explain the observed sex differences [21]. In addition, reference ranges for many biomarkers do not yet exist in the athlete population, which complicates their use for regular training load monitoring. Thus, the development of a reliable and exercise-sensitive panel of biomarkers for monitoring acute exercise responses is needed to gain a comprehensive understanding of immune responses to exercise. This may help to elucidate the complex interplay between biological sex, hormonal variations, and exercise responses in athletes, ultimately advancing the knowledge in this field.

The aim of the present study was to examine the acute effects of exhaustive exercise on various biomarkers (inflammatory cytokines, blood count, cfDNA, CK, and urea) in well-trained endurance athletes, with a focus on biological sex differences. This will be achieved by using exploratory bioinformatic analyses (e.g., mixed analysis of variance (ANOVA), k-means clustering, and uniform manifold approximation and projection (UMAP)) to provide novel insights into the relationships between sex, biomarkers, and exercise, ultimately contributing to the development of sex-specific biomarker assessments. In addition, we examined the subjective exercise response using questionnaires to assess fatigue, vitality, motivation, and energy. We hypothesized that blood markers would show a significant response to the exercise test, with differential effects between sexes.

## MATERIALS AND METHODS

### Ethics and experimental design

The study was registered (ClinicalTrials.gov identifier: NCT05067426). All procedures have been approved by a local ethical board (University of Salzburg, GZ 2/2021) and conform to the standards of the Declaration of Helsinki. Participants were informed of the risks and benefits of study participation and gave written informed consent. The data described in the present manuscript were collected during exercise testing [22]. Before and after exercise testing, participants underwent venous blood sampling and completed questionnaires.

### Participants

Twenty-four (16 men and 8 women) endurance-trained athletes were recruited. All participants had been involved in regular endurance training (running, trail running, triathlon, canoeing, biking, and soccer), completing an average of 4.7 ± 1.4 endurance training sessions per week. Participants’ characteristics are presented in Table 1.

**TABLE 1:** Anthropometric data and physiological data of the exercise testing (mean ± SD).

Variables	Overall (n = 24)	Men (n = 16)	Women (n = 8)
*Age (years)*	28.4 ± 7.1	29.2 ± 7.6**	26.8 ± 6.1
*Height (cm)*	177 ± 9	181 ± 7***	169 ± 6
*Weight (kg)*	69.8 ± 10.9	74.9 ± 7.8***	59.5 ± 9.0
*BMI (kg/m^2^)*	22.2 ± 2.2	22.9 ± 1.8*	20.9 ± 2.4
*Body Fat (%)*	11.8 ± 5.6	9.3 ± 3.2**	16.7 ± 6.3
$\dot{V}O_{2max}$ *(ml · min^−1^ · kg^−1^)*	57.2 ± 5.4	59.4 ± 7.5**	52.9 ± 3.1
*HR_max_ (bpm)*	190 ± 9	191 ± 10	187 ± 8
*RPE (AU)*	19.0 ± 0.6	19.1 ± 0.6	19 ± 0.5
*RER (AU)*	1.20 ± 0.06	1.21 ± 0.04	1.19 ± 0.08
*peak LA (mmol · L^−1^)*	9.7 ± 2.4	10.4 ± 1.7*	8.1 ± 2.8
*PPO (W)*	398 ± 83	445 ± 41***	300 ± 52
*Relative PPO (W · kg^−1^)*	5.7 ± 0.7	6.0 ± 0.5***	5.0 ± 0.4
*TTE total (min:sec)*	26:35 ± 2:07	26:25 ± 2:15	26:56 ± 1:54
*TTE ramp (min:sec)*	6:23 ± 1:07	6:34 ± 1:07	5:59 ± 1:05
*HR at LT (bpm)*	159 ± 9	158 ± 9	162 ± 7
*LT (km/h)*	12.2 ± 1.2	12.3 ± 1.3	12.0 ± 1.2

BMI, body mass index; ${\dot{\text{V}}\text{O}}_{2\max}$, maximal oxygen uptake during ramp test; HR max, maximal heart rate; RPE, rate of perceived exhaustion; RER, respiratory exchange rate; AU, arbitrary units; LA, lactate; PPO, peak power output during ramp test; TTE, time to exhaustion; LT, lactate threshold during submaximal exercise test;

* p < 0.05,

** p < 0.01,

*** p < 0.001 indicate significant differences in men and women.

### Physiological exercise testing

Prior to exercise testing, participants were instructed to refrain from strenuous exercise, alcohol, and caffeine for at least 24 h. Endurance performance was tested with a two-phase test on a treadmill (Saturn, HP Cosmos, Traunstein, Germany) with a breath-by-breath gas collection system (Quark CPET, Cosmed, Rome, Italy) [22]. Briefly, participants performed an incremental submaximal running test (with increases of 1.5 km/h every 3 min), followed by an 8 min recovery period, and a running ramp test until voluntary exhaustion on a treadmill. Heart rate (HR) was measured during the treadmill tests via a HR chest strap (HRM 3-SS, Kansas City, MO, USA). Running peak power output (PPO) was measured with the Stryd footpod (Stryd Wind V3, Stryd, Boulder, CO, USA) in absolute (W) and relative (W · kg^−1^) terms. Lactate was measured from capillary blood taken from the earlobe (Biosen S-line Clinic, EKF diagnostic GmbH, Magdeburg, Germany) throughout the incremental test. Peak LA was determined as the highest concentration collected immediately, five minutes, and 15 minutes after the ramp test. Rating of perceived exertion (RPE) on a scale from 6 to 20 [23] was collected immediately after completion of the ramp test. ${\dot{\text{V}}\text{O}}_{2\max}$ was determined as the highest 10 s breath rolling average. Analysis of further parameters such as lactate threshold (LT) and total time of test duration were determined, as described elsewhere [22], and are listed in Table 1.

### Blood parameters

Venous blood samples (3 ml EDTA and 3.5 ml serum) were obtained from the antecubital vein prior to the exercise test in a fasted condition and immediate after the ramp test. Venous blood count, i.e., white blood cell count (WBC), red blood cell count (RBC), absolute lymphocytes (LYM), percentage of lymphocytes (LYM%), absolute monocytes (MO), percentage of monocytes (MO%), absolute granulocytes (GR), percentage of granulocytes (GR%), procalcitonin (PCT), platelet (PLT), hemoglobin (HGB), hematocrit (HCT), red blood cell distribution width (RDWCV), mean platelet volume (MPV), mean corpuscular hemoglobin (MCH), mean corpuscular hemoglobin concentration (MCHC), mean corpuscular volume (MCV), and platelet distribution width (PDW) were determined using fresh whole blood by a Celltac MEK 6400 system (Nihon Kohden, Tokyo, Japan). EDTA samples were centrifuged at 1600 × g for 10 min at 4 °C, and serum samples were centrifuged at 3000 × g for 10 min at 4 °C. Samples were separated into aliquots and stored at ≤ − 20°C until further analysis.

Multiple cytokines (IL-2, IL-4, IL-5, IL-6, IL-9, IL-10, IL-13, IL-17A, IL-17F, IL-22, TNF-α, interferon-gamma (INF-γ)), related to different T-helper cell types were analyzed simultaneously, in duplicates, using a bead-based immunoassay multiplex approach (LEG-ENDplexTM HU Th Cytokine Panel (12-plex), BioLegend, San Diego, California, U.S) and was measured via flow cytometry (CytoFLEX S, Beckman Coulter, Brea, California, U.S). All steps were performed according to the manufacturer´s protocol with some minor changes: (i) downscaling of used kit components to a fifth based on a preliminary titration of the standard (ii) and adding 50 µl of individual sera without pre dilution. Venous cfDNA (cfDNA with 90 and 222 base pairs) was quantified by analyzing unpurified plasma via quantitative real-time PCR, as described elsewhere [24]. CK and urea were measured with a light emitting diode photometer (Eurolyser CCA180, Eurolyser Diagnostica GmbH, Salzburg, Austria).

### Vitality and fatigue assessment

Questionnaires were administered prior (pre) to exercise testing and in the evening between 6 PM and 8 PM (post). The modified German Subjective Vitality Scale (SVS-GM) with the 3-item (“I feel alive and vital”, “I am full of drive”, “I have energy and spirit”), as well as the 1-item (“I feel vital, full of drive, and spirited”) version were assessed on an 11-point Likert scale (0 = not true at all to 10 = totally true) [25] to measure perception of vitality, motivation, and energy. In addition, fatigue was assessed on an 11-point Likert scale (0 = not fatigued at all to 10 = total fatigue and exhaustion – nothing left) [26].

### Statistical analysis

Data are reported as means ± standard deviation (SD), while fold-changes from pre- to post-exercise are presented as median (all blood parameters; Tables 2 and 3) or mean fold-changes and 95% confidence intervals (CI) (all questionnaires; Table 4). To investigate the effects of exercise testing on blood parameters, questionnaire scores, and to identify differences between sexes in the physiological data of exercise testing (Table 1), pairwise t-tests were conducted for normally distributed data, and Wilcoxon-Tests for non-normally distributed data. Time × sex interactions were determined using mixed ANOVA. Effect sizes are reported as partial eta squared (_p_η^2^; small: |0.01| ≤ _p_η^2^ < |0.06|; medium: |0.06| ≤ _p_η^2^ < |0.14|; large _p_η^2^ ≥ |0.14|) [27]. The significance level was set at p < 0.05. The analyses were performed using IBM SPSS Statistics 26 (IBM GmbH, Munich, Germany).

**TABLE 2:** Acute changes in cfDNA and blood count.

Overall							Men					Women				ANOVA

Variable	Test	Pre	Post	FC (95% CI)	p-value	N	Pre	Post	FC (95% CI)	p-value	N	Pre	Post	FC (95% CI)	p-value	Time × Sex (p-value)
*cfDNA^90^ (ng/ml)*	Pairwise T-Test	9.3 (3.6)	103.2 (49.1)	11.3 (9.4 to 13.6)	**< 0.001**	16	9.4 (3.9)	117.8 (49.6)	12.5 (10.3 to 15.6)	**< 0.001**	8	8.9 (2.8)	74.1 (34.3)	7.2 (5.2 to 12.1)	**< 0.001**	**0.031**

*cfDNA^222^ (ng/ml)*	Pairwise T-Test	5.0 (1.7)	52.1 (24.8)	10.8 (8.9 to 12.2)	**< 0.001**	15	5.4 (1.6)	59.7 (25.6)	11.6 (9.1 to 13.5)	**< 0.001**	8	4.4 (1.8)	37.8 (16.1)	8.7 (6.4 to 11.8)	**< 0.001**	**0.044**

*LYM (10^3^/µl)*	Pairwise T-Test	1.5 (0.4)	3.8 (0.8)	2.7 (2.4 to 3.0)	**< 0.001**	16	1.5 (0.5)	3.6 (0.7)	2.6 (2.3 to 3.0)	**< 0.001**	8	1.5 (0.4)	4.2 (0.7)	3.1 (2.5 to 3.5)	**< 0.001**	**0.016**

*MO (10^3^/µl)*	Pairwise T-Test	0.4 (0.2)	0.9 (0.3)	2.3 (2.1 to 2.9)	**< 0.001**	16	0.4 (0.2)	0.8 (0.2)	2.3 (1.9 to 2.9)	**< 0.001**	8	0.5 (0.2)	1.1 (0.4)	2.5 (1.8 to 3.5)	**< 0.001**	0.094

*WBC (10^3^/µl)*	Pairwise T-Test	4.8 (1.0)	10.0 (1.8)	2.0 (2.0 to 2.3)	**< 0.001**	16	4.7 (1.0)	9.5 (1.8)	1.9 (1.9 to 2.2)	**< 0.001**	8	5.0 (1.0)	11.1 (1.4)	2.4 (1.9 to 2.6)	**< 0.001**	**0.038**

*GR (10^3^/µl)*	Pairwise T-Test	2.9 (0.9)	5.3 (1.2)	1.8 (1.7 to 2.0)	**< 0.001**	14	2.9 (0.7)	5.1 (1.05)	1.7 (1.6 to 2.0)	**< 0.001**	8	3.0 (1.2)	5.8 (1.4)	2.0 (1.6 to 2.4)	**< 0.001**	0.172

*CK (U/L)*	Wilcoxon-Test	203.3 (231.4)	306.2 (365.5)	1.4 (1.4 to 1.6)	**< 0.001**	13	192.7 (185.7)	292.5 (287.8)	1.6 (1.4 to 1.7)	0.005	7	222.8 (316)	331.4 (506.2)	1.4 (1.3 to 1.6)	0.183	0.896

*PCT (%)*	Wilcoxon-Test	0.1 (0.03)	0.2 (0.04)	1.4 (1.4 to 1.6)	**< 0.001**	16	0.1 (0.04)	0.2 (0.05)	1.4 (1.4 to 1.6)	**< 0.001**	8	0.1 (0.02)	0.2 (0.1)	1.3 (1.3 to 1.4)	**< 0.001**	**0.028**

*PLT (10^3^/µl)*	Wilcoxon-Test	202.1 (37.2)	279.0 (38.5)	1.4 (1.3 to 1.5)	**< 0.001**	16	202.0 (43.2)	281.1 (43.3)	1.4 (1.3 to 1.5)	**< 0.001**	8	200.3 (24.7)	274.6 (31.2)	1.3 (1.3 to 1.4)	**< 0.001**	0.57

*LYM (%)*	Pairwise T-Test	30.6 (7.5)	37.8 (5.8)	1.3 (1.2 to 1.4)	**< 0.001**	16	30.9 (7.0)	37.6 (6.1)	1.3 (1.2 to 1.3)	**< 0.001**	8	30.0 (8.8)	38.1 (5.5)	1.3 (1.1 to 1.5)	0.001	0.443

*MO (%)*	Pairwise T-Test	8.7 (3.6)	9.5 (2.9)	1.2 (1.0 to 1.5)	0.166	16	8.0 (2.7)	9.1 (2.4)	1.2 (1.0 to 1.5)	0.178	8	10.2 (4.9)	10.2 (3.9)	1.2 (0.7 to 1.6)	0.968	0.445

*HGB (g/dl)*	Pairwise T-Test	14.2 (1.2)	15.8 (1.0)	1.1 (1.1 to 1.1)	**< 0.001**	16	14.7 (0.8)	16.3 (0.6)	1.1 (1.1 to 1.1)	**< 0.001**	8	13.3 (1.2)	15.0 (1.0)	1.1 (1.1 to 1.2)	**< 0.001**	0.689

*HCT (%)*	Wilcoxon-Test	43.7 (3.1)	48.3 (3.6)	1.1 (1.1 to 1.1)	**< 0.001**	16	45.0 (1.8)	49.9 (1.4)	1.1 (1.1 to 1.1)	**< 0.001**	8	41.0 (3.5)	45.0 (4.4)	1.1 (1.1 to 1.1)	**< 0.001**	0.143

*RBC (10⁶/µl)*	Wilcoxon-Test	4.9 (0.3)	5.3 (0.4)	1.1 (1.1 to 1.1)	**< 0.001**	16	5.0 (0.2)	5.5 (0.3)	1.1 (1.1 to 1.1)	**< 0.001**	8	4.6 (0.4)	5.0 (0.5)	1.1 (1.0 to 1.1)	**< 0.001**	0.233

*RDWCV (%)*	Pairwise T-Test	12.2 (0.5)	12.6 (0.6)	1 (1.0 to 1.1)	**< 0.001**	16	12.1 (0.5)	12.5 (0.5)	1.0 (1.0 to 1.1)	0.444	8	12.3 (0.6)	12.9 (0.6)	1.0 (1.0 to 1.1)	0.191	0.396

*MPV (fL)*	Wilcoxon-Test	6.1 (1.4)	6.3 (1.3)	1 (1.0 to 1.1)	0.209	16	6.2 (1.5)	6.4 (1.4)	1.0 (1.0 to 1.1)	0.074	8	6.0 (1.2)	5.9 (1.1)	1.0 (0.9 to 1.0)	0.664	0.149

*MCH (pg)*	Pairwise T-Test	29.3 (2.0)	29.7 (1.9)	1 (1.0 to 1.0)	**< 0.001**	16	29.6 (2.0)	30.0 (1.9)	1.0 (1.0 to 1.0)	**0.003**	8	28.5 (2.1)	29.1 (2.0)	1.0 (1.0 to 1.0)	**0.007**	0.508

*Urea (mg/dl)*	Wilcoxon-Test	32.4 (9.4)	32.6 (8.5)	1 (0.9 to 1.1)	0.627	13	34.4 (9.7)	34.5 (7.7)	1.0 (0.9 to 1.2)	0.928	7	28.9 (8.4)	29.0 (9.3)	1.0 (0.9 to 1.1)	0.916	0.981

*MCHC (g/dl)*	Wilcoxon-Test	32.6 (0.7)	32.6 (0.7)	1 (1.0 to 1.0)	0.796	16	32.6 (0.8)	32.7 (0.8)	1.0 (1.0 to 1.0)	0.815	8	32.3 (0.8)	32.5 (0.7)	1.0 (1.0 to 1.0)	0.53	0.714

*MCV (fl)*	Pairwise T-Test	89.8 (4.8)	90.9 (4.8)	1 (1.0 to 1.0)	**< 0.001**	16	90.6 (4.9)	91.7 (4.9)	1.0 (1.0 to 1.0)	**< 0.001**	8	88.4 (4.6)	89.4 (4.5)	1.0 (1.0 to 1.0)	**0.002**	0.709

*PDW (%)*	Wilcoxon-Test	17.2 (1.7)	17.3 (1.9)	1 (0.9 to 1.0)	0.455	16	16.8 (1.9)	16.8 (2.1)	1.0 (1.0 to 1.0)	0.986	8	17.9 (0.8)	18.1 (1.3)	1.0 (0.9 to 1.1)	0.686	0.734

*GR (%)*	Pairwise T-Test	61.5 (9.7)	55.0 (10.0)	0.9 (0.8 to 0.9)	**< 0.001**	14	62.4 (7.4)	57.0 (10.4)	0.9 (0.9 to 1.0)	**0.015**	8	59.9 (13.3)	51.7 (9.1)	0.8 (0.8 to 1.0)	**0.018**	0.389

Pre and post values are shown in mean and SD; pre, before exercise test; post, immediate after exercise test; FC, median fold change; 95% CI, 95% confidence interval; N, number of participants; time × sex ANOVA; cfDNA, cell-free DNA; WBC, white blood cell count; RBC, red blood cell count; LYM, lymphocytes; MO, monocytes; GR, absolute granulocytes; CK, creatine kinase; PCT, procalcitonin; PLT, platelet; HGB, hemoglobin; HCT, hematocrit; RDWCV, red blood cell distribution width; MPV, mean platelet volume; MCH, mean corpuscular hemoglobin; MCHC, mean corpuscular hemoglobin concentration; MCV, mean corpuscular volume; PDW, platelet distribution width; highlighted are significant p values < 0.05. N differences are due to analysis.

**TABLE 3:** Acute changes in cytokines.

Overall							Men					Women				ANOVA

Variable	Test	Pre	Post	FC (95% CI)	p-value	N	Pre	Post	FC (95% CI)	p-value	N	Pre	Post	FC (95% CI)	p-value	Time × Sex
*IL-6 (pg/ml)*	Pairwise T-Test	29.5 (21.8)	36.7 (21.4)	1.4 (0.9 to 2.6)	0.082	12	33.0 (26.1)	44.0 (23.8)	1.4 (0.7 to 3.5)	**0.038**	8	24.1 (13.2)	25.7 (10.9)	1.3 (0.7 to 1.8)	0.823	0.25

*IL-17α (pg/ml)*	Wilcoxon-Test	7.8 (7.0)	6.8 (5.1)	1.3 (0.9 to 1.6)	0.91	11	6.5 (7.0)	5.9 (4.8)	1.4 (0.9 to 1.9)	0.737	4	11.3 (6.5)	9.4 (5.5)	0.9 (0.6 to 1.1)	0.121	0.659

*IL-17F (pg/ml)*	Pairwise T-Test	253.2 (325.8)	255.7 (309.1)	1.2 (0.4 to 3.4)	0.794	12	276.9 (389.9)	287.0 (370.5)	1.3 (-0.6 to 4.6)	0.399	6	205.8 (150.2)	193.2 (126.1)	1.0 (0.7 to 1.3)	0.475	0.271

*IL-4 (pg/ml)*	Wilcoxon-Test	85.4 (119.8)	82.3 (124.2)	1.2 (1.0 to 1.8)	0.236	15	56.6 (76.6)	60.6 (62.3)	1.2 (1.7 to 2.1)	0.631	8	139.5 (168.0)	123.0 (194.5)	1.0 (0.5 to 1.5)	0.652	0.464

*IL-22 (pg/ml)*	Pairwise T-Test	12.6 (9.2)	13.3 (8.1)	1.1 (0.9 to 1.7)	0.679	11	12.9 (10.1)	14.0 (8.2)	1.0 (0.9 to 1.9)	0.556	5	12.0 (8.0)	11.7 (8.6)	1.1 (0.3 to 2.2)	0.931	0.692

*IL-2 (pg/ml)*	Wilcoxon-Test	4.2 (3.5)	4.2 (3.0)	1.1 (1.0 to 1.4)	0.08	11	3.9 (3.1)	4.4 (3.0)	1.1 (1.0 to 1.4)	**0.027**	8	4.7 (4.2)	3.8 (3.1)	1.1 (0.7 to 1.6)	0.492	0.197

*IL-9 (pg/ml)*	Wilcoxon-Test	33.4 (29.7)	33.7 (27.8)	1.1 (1.0 to 1.2)	0.094	15	31.5 (27.9)	33.1 (25.6)	1.1 (1.0 to 1.3)	0.147	8	37.1 (34.5)	34.9 (33.4)	1.0 (0.8 to 1.3)	0.716	0.393

*IL-10 (pg/ml)*	Wilcoxon-Test	6.2 (3.8)	6.1 (3.5)	1.1 (0.9 to 1.2)	0.566	13	6.1 (4.3)	6.1 (3.6)	1.0 (0.9 to 1.2)	0.975	8	6.3 (3.3)	6.1 (3.6)	1.1 (0.7 to 1.5)	0.938	0.916

*TNF-α (pg/ml)*	Pairwise T-Test	49.6 (37.5)	47.1 (32.8)	1.0 (0.8 to 1.5)	0.424	11	48.3 (45.1)	49.0 (39.4)	1.1 (0.8 to 1.9)	0.831	5	52.5 (13.0)	43.0 (11.2)	0.9 (0.6 to 1.1)	0.203	0.124

*IFN-γ (pg/ml)*	Pairwise T-Test	66.5 (59.1)	72.1 (58.5)	1.0 (0.5 to 2.7)	0.364	8	80.8 (72.5)	84.6 (71.2)	1.0 (-0.1 to 3.8)	0.602	5	56.6 (23.7)	66.0 (25.6)	1.1 (0.6 to 2.0)	0.511	0.684

*IL-13 (pg/ml)*	Wilcoxon-Test	27.7 (22.1)	22.9 (15.2)	1.0 (0.7 to 1.3)	0.433	9	26.9 (22.1)	25.6 (16.0)	1.1 (0.8 to 1.5)	0.69	5	29.2 (24.6)	18.0 (14.1)	0.5 (0.1 to 1.3)	0.267	0.228

*IL-5 (pg/ml)*	Wilcoxon-Test	20.7 (17.2)	18.5 (12.3)	0.9 (0.8 to 1.2)	0.36	12	23.9 (20.2)	20.2 (14.6)	0.9 (0.9 to 1.1)	0.209	6	14.3 (6.3)	15.3 (4.9)	1.1 (0.5 to 2.0)	0.76	0.313

Pre and post values are shown in mean and SD; pre, before exercise test; post, immediate after exercise test; FC, median fold change; 95% CI, 95% confidence interval; N, number of participants; time × sex ANOVA; IL, interleukin; TNF-α, tumor necrosis factor-alpha; highlighted are significant p values < 0.05. N differences are due to analysis.

To ensure mathematical comparability for unsupervised machine learning, all continuous variables were normalized and scaled using min-max scaling in R Studio (R Studio Inc., Boston, MA, United States, version 4.2.1). Missing values (i.e., cytokine levels that were below or exceeded the standard curve) accounted for 9% of the data. Using the Mice package (version 3.15.0), these missing values were imputed [28]. The “umap” package (version 0.92) was used to construct a UMAP – a technique for visualizing high-dimensional data in two dimensions, and subsequent control-correlations were performed to compare athletes versus biomarkers (Figure 3B, Supplement Figure A [29]).

For K-means unsupervised clustering, the “mlr3” package (version 2.19.1) was used [30]. Cytokines and blood markers were used relative to absolute values. This decision was necessary because relative values reduce the risk of overfitting, which can occur when the number of variables exceeds the number of observations. In addition, relative values account for differences in baseline values, inter-individual variances and better reflect each athlete’s physiological status than absolute numbers. Finally, relative values can limit the impact of extraneous factors like time of collection or modest methodological variances, which may affect absolute levels but not relative changes. The optimal number of clusters was determined using a cluster screening technique, which suggested that three clusters provided the best fit to the model, and three clusters were chosen for further analysis (Supplement Figure B). Participants belonging to each cluster and the weight of importance of each biomarker for each cluster were obtained (Figure 3A).

**FIG. 1:**
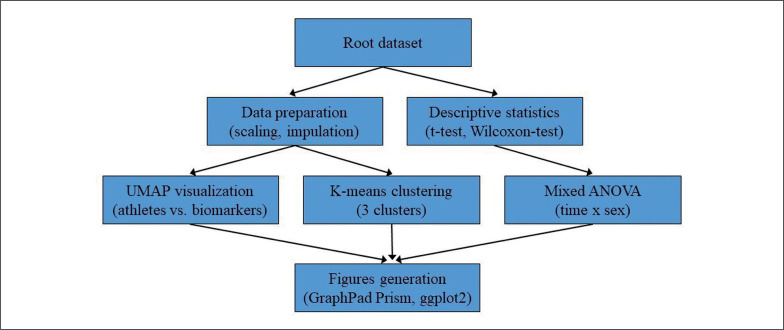
Flowchart illustrating the statistical analysis process employed in the study. The root dataset was used for both data preparation (scaling and imputation) and descriptive statistics (t-tests and Wilcoxon-Test). Data preparation was followed by UMAP visualization and K-means clustering, while descriptive statistics were followed by mixed ANOVA analysis. The results from the mixed ANOVA, UMAP visualization, and K-means clustering were then used to generate figures using GraphPad Prism and ggplot2 in R.

Figures were generated using GraphPad Prism version 9 (Prism, GraphPad Software, San Diego, CA, USA) and the package ggplot2 3.2.0 (R Studio Inc., Boston, MA, United States, version 4.2.1). The overall statistical analysis process is illustrated in Figure 1.

## RESULTS

Table 1 presents the characteristics of physiological data collected from the 24 participants during exercise testing. Mean ${\dot{\text{V}}\text{O}}_{2\max}$ was 57.2 ± 5.4 ml · min^−1^ · kg^−1^, with men showing a slightly higher value compared to women on both outcomes (p < 0.01). The mean maximum LA concentration was 9.7 ± 2.4 mmol · L^−1^ for all participants with higher concentrations in men compared to women (p < 0.05). Mean PPO was 398 ± 83 W, and the relative PPO was 5.7 ± 0.7 W · kg^−1^ for all participants, with men showing higher values compared to women (p < 0.001). Other physiological characteristics showed no significant differences between the sexes.

**FIG. 2:**
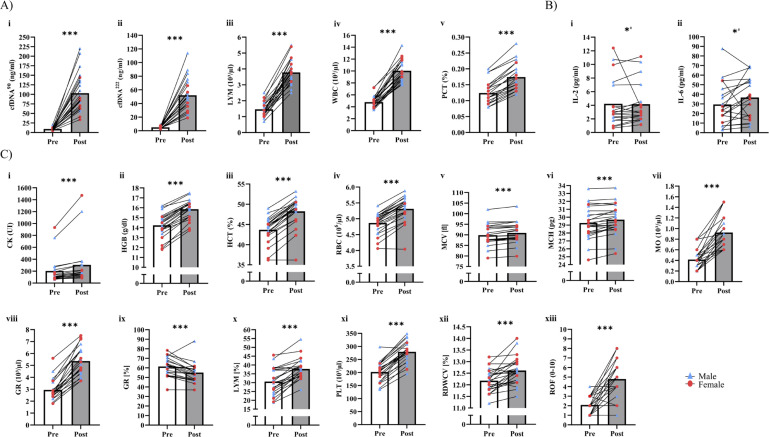
Acute increases of blood-based biomarkers and questionnaires. A) Blood-based biomarkers with significant time × sex ANOVA. B) Female vs. male acute increase of cytokines. C) Overall acute increases of blood-based biomarkers and questionnaires. Red dots, female participants; blue triangle, male participants; bars represent mean values; white bar, pre; grey bar, immediate post-exercise; CK, creatine kinase; WBC, white blood cell count; RBC, red blood cell count; HGB, hemoglobin; HCT, hematocrit; MCV, mean corpuscular volume; MCH, mean corpuscular hemoglobin; PLT, platelet; LYM, lymphocytes; MO, monocytes; GR, absolute granulocytes; RDWCV, red blood cell distribution width; PCT, procalcitonin; ROF, rate of fatigue; * p < 0.05, *** p < 0.001, # male.

Table 2 outlines time × sex interactions in biomarker concentrations. Significant interactions were observed for cfDNA^90^ (p = 0.031, _p_η^2^ = 0.20), cfDNA^222^ (p = 0.044, _p_η^2^ = 0.18), and PCT (p = 0.028, _p_η^2^ = 0.20). In these cases, male participants had higher fold-changes than female participants (cfDNA^90^: 12.5 vs. 7.2, cfDNA^222^: 11.6 vs. 8.7, PCT: 1.4 vs. 1.3). In addition, significant interactions were found in LYM (p = 0.016, _p_η^2^ = 0.24) and WBC (p = 0.038, _p_η^2^ = 0.18), with female participants showing higher fold-changes than male participants (LYM: 3.1 vs. 2.6, WBC: 2.4 vs. 1.9; Figure 2A i-v). No significant interactions were found for all cytokines (p > 0.05).

Tables 2 and 3 present the acute effects of exercise testing on biomarker concentrations. Significant increases were found for both the overall population, as well as male and female participants, in cfDNA^90^, cfDNA^222^, LYM, LYM%, MO, WBC, GR, GR%, PCT, PLT, HGB, HCT, RBC, MCH, MCV (p < 0.01; Figure 2C i-xiii). RDWCV and CK increased in all participants (p < 0.001), as well as CK in male participants (p = 0.005). IL-2 (p = 0.027) and IL-6 (p = 0.038) increased in male participants, while no changes in the female participants were found (Figure 2B i, ii). Other cytokines showed no significant differences (p > 0.05). Individual changes for all significantly increased variables are shown in Figure 2.

Table 4 presents the effects of exercise testing on vitality and fatigue data. Neither changes nor interactions between time and sex were found in vitality, motivation, and energy. However, fatigue increased overall (p < 0.001), but no interactions of time × sex were identified (p = 0.334; Figure 2C xii).

**TABLE 4:** Acute changes in vitality and fatigue.

Overall							Men					Women				ANOVA

Variable	Test	Pre	Post	FC (95% CI)	p-value	N	Pre	Post	FC (95% CI)	p-value	N	Pre	Post	FC (95% CI)	p-value	Time × Sex
*Fatigue*	Pairwise T-Test	2.1 (0.9)	4.8 (1.8)	2.4 (0.6 to 1.4)	**< 0.001**	16	1.9 (0.9)	4.4 (1.6)	2.4 (1.9 to 3.6)	**< 0.001**	8	2.4 (0.9)	5.6 (1.9)	2.5 (1.2 to 4.7)	**0.005**	0.334

*SVS-GM-3 vitality*	Pairwise T-Test	6.9 (1.7)	6.5 (1.5)	0.9 (0.8 to 1.2)	0.375	16	7.1 (1.6)	6.9 (1.1)	0.9 (0.9 to 1.2)	0.615	8	6.5 (2.0)	5.6 (1.9)	0.9 (0.6 to 1.4)	0.495	0.573

*SVS-GM-3 motivation*	Pairwise T-Test	7.1 (1.8)	5.9 (1.8)	0.9 (0.7 to 1.1)	0.05	16	7.1 (1.7)	6.2 (1.2)	0.9 (0.8 to 1.1)	0.095	8	7.1 (2.1)	5.3 (2.7)	0.8 (0.3 to 1.4)	0.25	0.431

*SVS-GM-3 energy*	Pairwise T-Test	7.3 (1.9)	6.3 (1.8)	0.9 (0.8 to 1.1)	0.079	16	7.5 (1.8)	6.6 (1.3)	0.8 (0.8 to 1.1)	0.084	8	7.0 (2.1)	6.6 (1.3)	1.0 (0.4 to 1.4)	0.368	0.628

*SVS-GM-1 overall*	Pairwise T-Test	7.1 (1.5)	6.1 (1.7)	0.9 (0.8 to 1.1)	0.062	16	7.4 (1.3)	6.5 (1.3)	0.9 (0.8 to 1.0)	0.063	8	6.6 (1.9)	5.4 (2.2)	0.8 (0.4 to 1.4)	0.375	0.738

Pre and post values are shown in mean and SD; pre, before exercise test; post, evening post-exercise test; SVS-GM, 1-item, and 3-item modified German Subjective Vitality Scale; FC, mean fold change; 95% CI, 95% confidence interval; N, number of participants; time × sex ANOVA; highlighted are significant p values < 0.05.

**FIG. 3:**
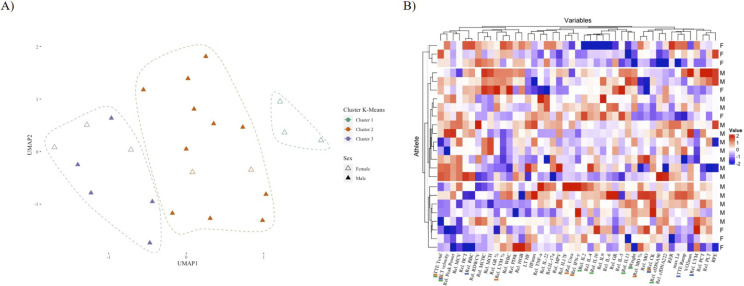
Characterization of three distinct athlete clusters based on key biomarkers. A) UMAP plot illustrating the distribution of athletes according to sex and k-means clustering with three clusters: “Cluster 1” (n = 3), “Cluster 2” (n = 13, 2 females), and “Cluster 3” (n = 8, 3 females). Female athletes are represented by unfilled triangles, while male athletes are represented by colored filled triangles. B) Heatmap displaying the standardized values of 46 variables (rows) for each athlete (columns), with row annotations indicating the sex (F: Female, M: Male) of each athlete. Clusters are color-coded according to the same scheme used in (A). The top markers are represented as colored bars in the names, being green (Cluster 1), red (Cluster 2), and blue (Cluster 3).The three clusters exhibit distinct patterns of biomarkers, with significant differences in their sex compositions (X-squared = 7.9471, df = 2, p-value = 0.02).

K-means clustering identified three distinct groups of athletes with differing proportions of female participants, which could be classified as “cluster 1” (n = 3, 100% female), “cluster 2” (n = 13, 85% male), and “cluster 3” (n = 8, 37.5% female and 65.5% male) (Figure 3A). The correlation revealed distinct patterns among the three groups of athletes (Figure 3B). Each cluster was distinguished by a particular combination of biomarkers and exercise testing performance parameters. Loadings derived from k-means clustering offer insights into the distribution of variables within each cluster and serve as descriptive summaries of the central tendencies of variables within each cluster (Supplement Figure C). Specifically, a positive loading for a particular variable within a cluster signifies that cluster members exhibit higher values for that variable in comparison to members of other clusters. Conversely, a negative loading implies the opposite.

Cluster 1 was characterized by negative loadings for weight, ${\dot{\text{V}}\text{O}}_{2\max}$, maximal HR (HR_max_), and relative PPO but a positive loading for RPE. Most of the inflammatory markers had negative loadings, suggesting lower levels of these markers in this cluster. This aligns with the observation that relative IL-2, relative IL-10, relative IL-13, relative cfDNA^90^, total TTE, LT, and body weight served as the primary markers for the cluster consisting of females, only. Cluster 2 had a negative loading for ${\dot{\text{V}}\text{O}}_{2\max}$ and relative PPO but a positive loading for HR_max_. The loadings for inflammatory markers were mixed, but many were positive, suggesting higher levels of these markers compared to cluster 1. This is characterized by relative MO, relative IFN-y, relative LYM, relative CK, relative urea, relative PPO, total TTE, and LT serving as the primary markers for the cluster consisting primarily of men. Cluster 3 exhibited predominantly positive loadings for most of the exercise testing performance parameters. In terms of the inflammatory markers, the loadings were mixed, with many showing a positive, indication a diverse immune response. Nevertheless, it is crucial to recognize the variability in sex distribution within this cluster. A chi-squared test was performed on the contingency table of clusters and sex, and the results showed a significant difference (X-squared = 7.9471, df = 2, p = 0.02).

To provide a comprehensive understanding of this process, a full description of the variable importance is included in the supplementary data, which is represented in the form of a heatmap of the centers (Supplement Figure C). This heatmap serves as a visual representation of the variables’ contribution to the k-means clustering and provides insight into their relative importance for each level of clustering (1, 2, and 3), rather than for the individual levels of the athletes’ variables.

## DISCUSSION

The present study examined established and novel biomarkers, such as various cytokines and cfDNA, in an acute exhaustive exercise setting with respect to sex differences. Acute increases in venous blood count markers and cfDNA were observed in male and female athletes, while cytokines were unaffected, except for IL-2 and IL-6, which showed larger increases in men. Male and female athletes exhibited similar acute responses in blood count markers. Cell-free DNA^222^ and cfDNA^90^ increased significantly in male and female athletes, with male athletes showing higher increases than their female counterparts. In contrast, post-exercise WBC and LYM concentrations were higher in female athletes. For questionnaires, only fatigue was affected by the exercise test. This study innovatively applies the k-means clustering method to detect potential distributions in biomarker profiles between biological sexes. Three distinct groups of athletes with varying proportions of female participants were identified. Notably, in the exclusively female cluster, relative changes in cytokines such as IL-2, IL-10, IL-13, and cfDNA^90^ were the primary markers that differed from the other clusters.

Cell-free DNA and cytokines have been increasingly adopted in studies of acute and chronic stress responses in recent years [31]. Particularly, cfDNA has demonstrated good reliability with marked acute responses during aerobic running [12], incremental testing [32], and intermittent exercise, even demonstrating a relationship with distance covered in soccer players [8]. In line with previous findings, cfDNA significantly increased after exercise testing, further highlighting cfDNA as an exercise responsive biomarker. For the first time, sex differences were shown for both cfDNA^90^ and cfDNA^222^ (Figure 2A i, ii, and Table 2). The more pronounced increases in cfDNA concentrations in males may be attributed to the longer duration of the exercise test, higher LA concentration, and a higher relative PPO (Table 2) [31]. Hormonal differences, including menstrual cycle fluctuations in women, could also contribute to sex differences in relative changes in cfDNA [33], although literature on this topic is scarce [34]. To our knowledge, only Pölcher et al. [34] have shown that cfDNA levels do not differ during the different phases of the menstrual cycle when studying healthy participants and cancer patients. Our study did not control for the menstrual cycle, which could have affected the immune response. Therefore, it remains to be determined which factors impact sex differences in cfDNA.

Previous studies have shown an increase in cytokine concentrations during prolonged running [11, 14] with athletes generally showing an attenuated response [3, 35]. Our results showed slight significant increases in IL-2 and IL-6 in males (Table 3), while other studies have demonstrated acute increases in other cytokines as well (e.g., TNF-α, IL-10, IL-1 receptor antagonist) [3, 10, 14, 18, 20, 35]. In particular, IL-6 has been identified as an exercise-sensitive bio-marker [18]. Lobo et al. [20] observed similar responses in IL-6 levels in females and males (2.8- and 2.3-fold) after a fatiguing aerobic exercise protocol, suggesting similar immunological responses between sexes. Conversely, we found a significant increase in IL-6 concentrations in male participants, while baseline values in females were lower and did not change significantly. Bernardi et al. [21] found higher baseline values in IL-6, IL-1β, and TNF-α in healthy males compared to females.

Whole blood count markers (e.g., MCV, HGB, PLT, RBC, HCT, and MCHC) have been shown to be reliable and sensitive to acute exercise [18]. This is consistent with our results, as 15 biomarkers were acutely elevated in male and female participants, as an exercise-induced stress response (Table 2). The highest overall increases (2.0–2.7-fold) were observed in leukocytes (i.e., WBC, LYM, MO) which is in line with other studies [10, 20, 36]. Our data disagrees with the findings from Lobo et al. [20] who reported similar changes in both sexes in WBC and LYM. However, the training protocol differed from the current protocol with participants having a lower training level compared to our participants [20]. Alis et al. [37] reported, concurring with our results, an increase in platelets after exhaustive exercise. Low HGB concentration in athletes, specifically in females can be to the results of iron deficiency [38], even though our results showed no acute differences in male and female participants. Conflicting results regarding sex differences and limited information for athletes persist about haematological markers and require further investigation [37].

Creatine kinase has been used in acute and chronic situations to monitor internal load and an immediate exercise-induced increase has been observed in various modes of exercise, in accordance with our data [39]. However, peak levels can be observed 48 hours to five days after exercise, and men generally have higher resting serum CK concentrations than women [40–42]. Similarly, in our study, CK levels immediately increased after the exercise in two participants who had higher baseline values, while others increased slightly (Figure 2B i). This shows that post-exercise CK levels have high variability, e.g., due to different types of responders [43, 44].

We observed no significant changes in subjective vitality, motivation, or energy (Table 4). However, motivation declined from morning to evening and reached borderline significance (p = 0.05). The original SVS by Ryan and Frederick [45] has shown to be influenced by long-term exercise, however, the SVS-GM has not been used in an acute exercise setting. Buchner et al. [25] reported an increase in vitality throughout the day in an everyday life scenario rather than an acute response. Hereby, it requires further investigation if lower exercise intensities than in our study lead to increases in subjective vitality. Fatigue increased significantly in male and female participants, reflecting the exhaustive nature of the exercise test (RPE > 19).

The study identified three distinct groups of athletes with differing proportions of female participants, which emphasizes the relevance of sex-specific biomarker profiles and individualized exercise response within athletic populations. In the exclusively female cluster, markers such as IL-2, IL-10, IL-13, and cfDNA^90^ were identified, suggesting the possibility of targeted interventions to modulate immune responses specifically in female athletes. In cluster 2 (primarily composed of males), relevant markers included MO, IFN-y, LYM, and CK, whereas cluster 3 (featured both males and females) exhibited RBC, LYM, and CK markers. Since previous studies have reported mixed findings regarding sex-related differences in exercise-induced immune responses [11, 14, 20, 21, 46, 47], it remains uncertain whether biological sex influenced the observed negative correlation between IL-2, IL-10, and IL-13 and female participants in cluster 1 (Supplement Figure C). In addition, our cluster classification accounts for all levels of cytokine distributions, which is essential for a comprehensive understanding of immune response. Similar to a study by Xiong et al. [48], where k-means clustering was employed as part of a machine learning approach, aiming to elucidate the distribution patterns of cytokines and their significance in the context of cytokine release syndrome detection. Despite prior research investigating biomarkers associated with exercise and physical performance, only one study has explored the potential differences in biomarker profiles based on biological sex and resulting clustering patterns [49].

Utilizing cluster analysis based on biological sex may provide insights into potential sex-related differences in biomarker response to exercise. Furthermore, the holistic examination of multiple biomarkers remains essential for a comprehensive understanding of immune responses to exercise. Through k-means clustering, we identified distinct patterns among the biomarkers. However, it is important to note that the small sample size may limit the generalizability of the findings. One inherent limitation of k-means clustering is its sensitivity to unbalanced group sizes. The algorithm aims to minimize withincluster variance, which can be dominated by larger clusters, potentially overshadowing smaller but significant clusters [50]. To address these limitations and improve the robustness of our analyses, a larger sample size with a more balanced sex distribution would be beneficial. This would enable more robust statistical analyses and further improve the machine learning predictions, enhancing the evaluation of the predictive power of the identified biomarkers. Consequently, replication studies on a larger sample size would advance work in this area.

Our observations are limited to two time points and do not indicate whether there are any cluster-specific effects in the kinetics of these biomarkers returning to baseline. In addition, the evaluation of the subjective assessment in the evening may have been influenced by uncontrolled factors (e.g., stress in personal and work life). This study did not control for the menstrual cycle phase of female participants, which could have impacted the biomarker concentrations. Investigating menstrual shifts in hormonal status could provide further detail regarding sex differences in the acute exercise response. Lastly, further analysis for biomarker sensitivity is essential as reference ranges for athletes are missing, and there are high intra-individual differences. In particular, the cytokine results showed a certain variability and data were missing because there was not a concentration detected for each cytokine.

## CONCLUSIONS

In summary, our results identified exercise-sensitive biomarkers (cfDNA^90^, cfDNA^222^, and blood count markers) for monitoring the acute exercise response. While sex differences were found in certain blood count markers and cfDNA, the k-means cluster analysis revealed three distinct groups with varying proportions of female participants and different cytokine levels. This suggests that investigating sex-specific cytokine profiles, particularly related to IL-2, IL-10, IL-13, and cfDNA^90^, may play a crucial role in exercise research and practice. Differences between women and men in certain biomarkers highlight the need for establishing a set of biomarkers for exercise testing and to further investigate sex differences in athletes.

## Supplementary Material

Exploring sex differences in blood-based biomarkers following exhaustive exercise using bioinformatics analysis

## Data Availability

The dataset used and analyzed during the current study is available from the corresponding author on reasonable request.
