# Supplementary material for: Exploring sex differences in blood-based biomarkers following exhaustive exercise using bioinformatics analysis
Source: Biol Sport. 2024 Jan 2;41(3):105–18. doi: 10.5114/biolsport.2024.132998 (PMC11167456; doi:10.5114/biolsport.2024.132998)
Supplement: Exploring sex differences in blood-based biomarkers following exhaustive exercise using bioinformatics analysis [file JBS-41-51826-s1.pdf]

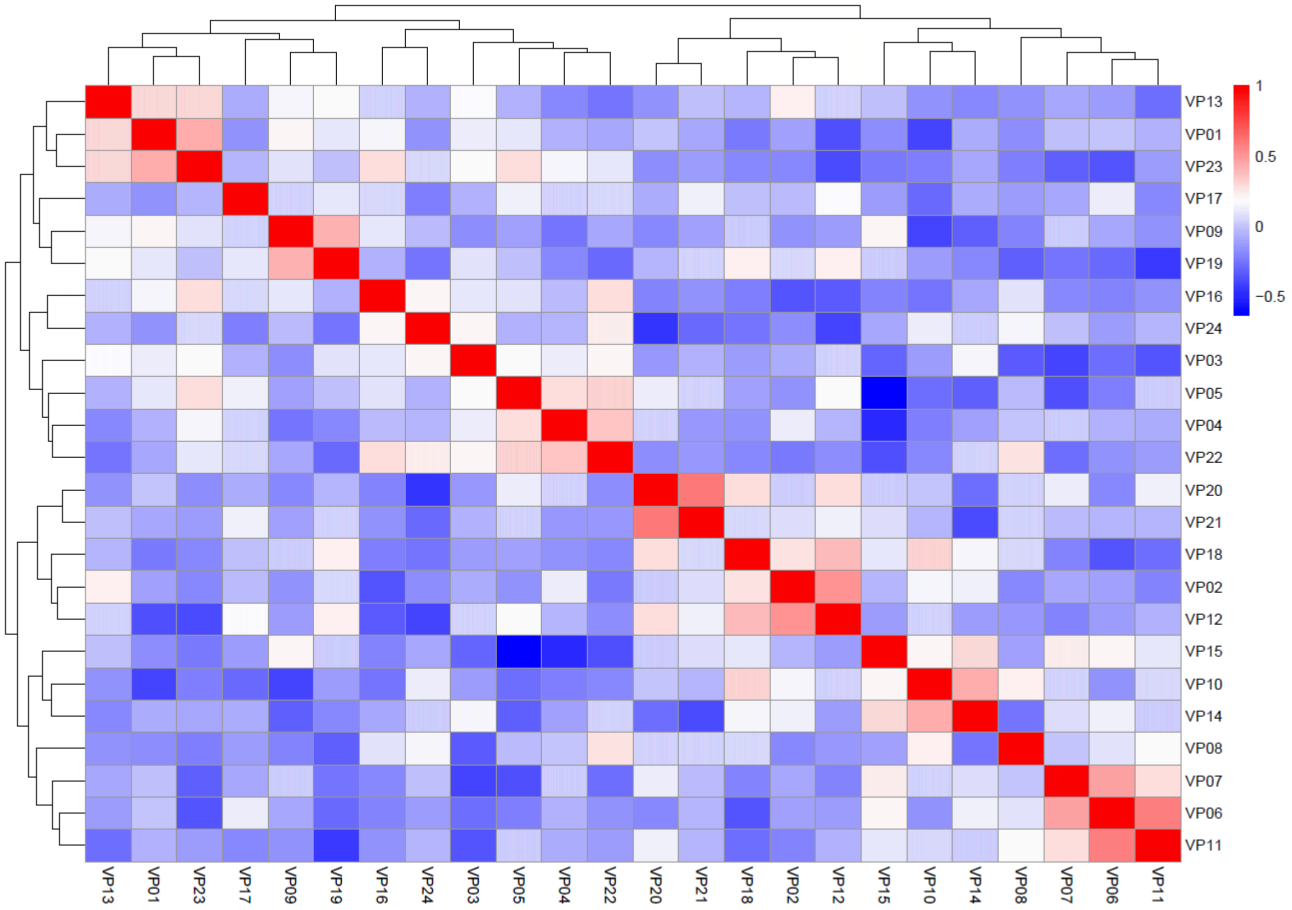

SUPPLEMENT FIGURE A: Heatmap of Spearman's correlation matrix of log-scaled data. This figure shows the heatmap of the Spearman's correlation matrix of the log-scaled data. The heatmap provides a visual representation of the correlations between each pair of variables, with red indicating positive correlations and blue indicating negative correlations.

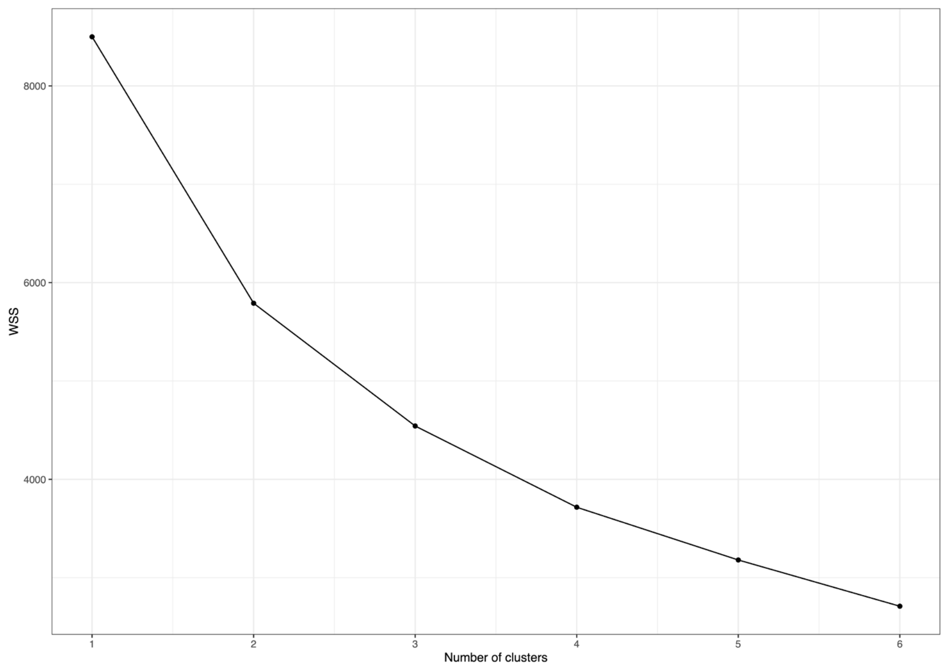

SUPPLEMENT FIGURE B: Plot of WSS versus number of clusters. This figure shows the plot of the Within-Cluster-Sum-of-Squares (WSS) versus the number of clusters in the K-means algorithm. The WSS is a measure of the sum of distances between data points and the centroid of their respective clusters. The plot provides a visual representation of how the WSS changes with the number of clusters, and can be used to determine the optimal number of clusters.

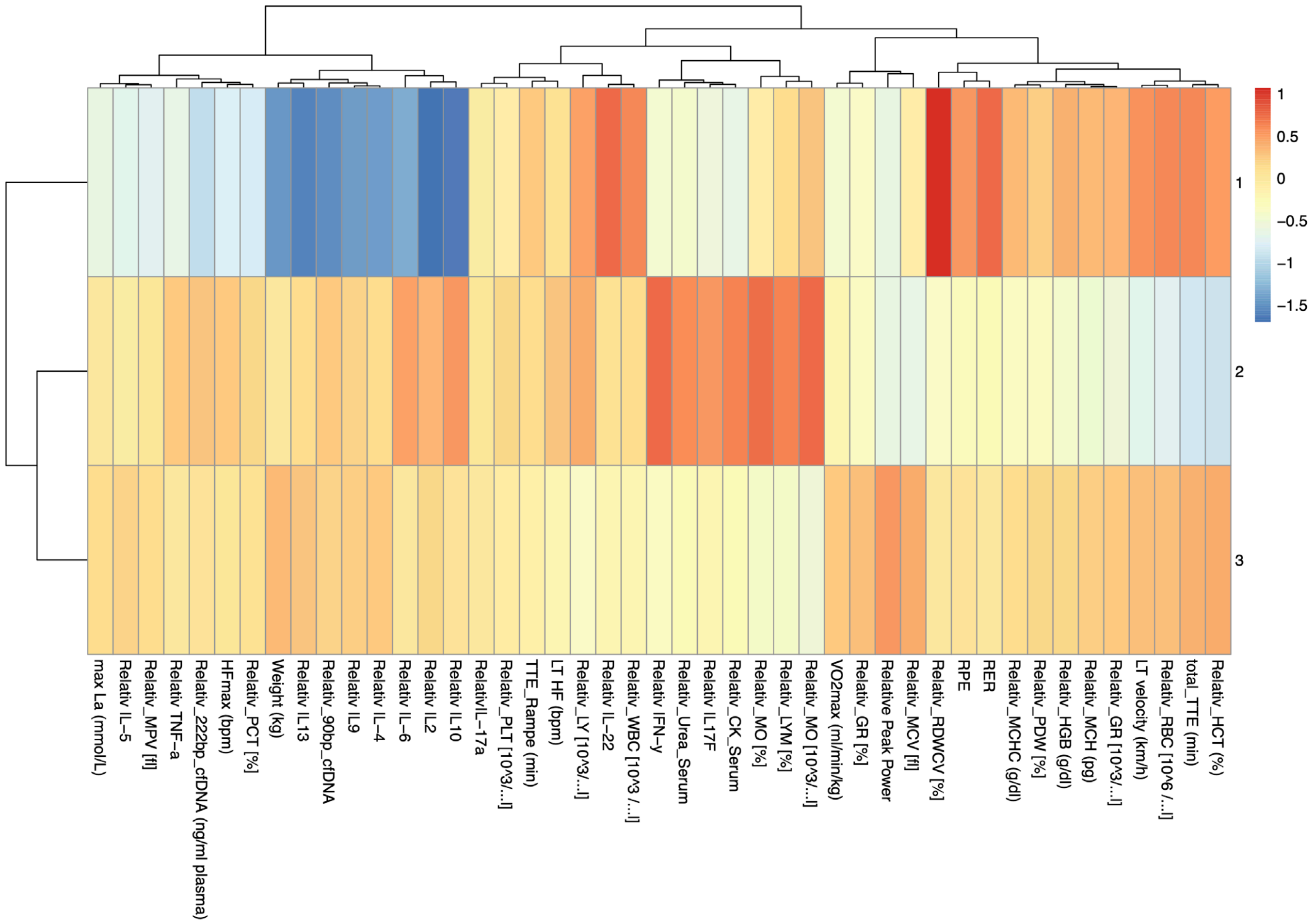

SUPPLEMENT FIGURE C: Heatmap of Cluster Centers. This figure shows the heatmap of the cluster centers after being clustered using the K-means algorithm with 3 clusters. The heatmap provides a visual representation of the cluster centers, which are the average of the data points in each cluster. The center values represent the importance of each variable in representing the cluster.
